# Knight in Common Armor: An Interview with Sir John Sulston

**DOI:** 10.1371/journal.pgen.0020225

**Published:** 2006-12-29

**Authors:** Jane Gitschier

I spent two of my three months on sabbatical in Cambridge gathering my courage to invite Sir John Sulston for an interview. How do you approach a man who has spearheaded and labored with his own hands on three major genetics projects, won a Nobel Prize, been knighted, and even had a building named after him? Perhaps it was the salutation that gave me pause, before settling finally on “dear sir john” in an e-mail.

Capped with a head of vigorous white hair and a face framed with a matching beard, Sir John has a rock star recognizability. Thus he captured my attention one Saturday afternoon as my 12-year-old daughter and I bicycled past the Fitzwilliam Museum, and he pedaled by in the opposite direction. “There is the man I'm hoping to interview!” I said as I pointed out the man in a red shirt.

I told her he was Sir John Sulston. “Is he a prince?” No, just a knight.

That he had won a Nobel Prize. “Is he rich?” I don't think so.

And that he had won it for his work on worms. “Does he know that worms are segmented?” Ah, a tricky question! Not his worms, tiny creatures called C. elegans. I started to wonder if maybe she should do the interview.

Sir John was catapulted into the public light as the spokesperson for the human genome in the UK. His experiences in defending the public genome efforts against the assault of privatization and patenting, chronicled in his book *The Common Thread: A Story of Science, Politics, Ethics and the Human Genome* (Black Swan, 2003) were transformational. Now officially retired from the Wellcome Trust Sanger Institute (WTSI), he is absorbed in policy-making for the UK government, the World Health Organization, and a variety of nongovernmental organizations (NGOs). An activist and humanitarian who still rides his bicycle to work, who is content to share a small office, and who ponders whether our species will survive the coming century, Sir John is an inspirational man, one who leads by example.

**Jane Gitschier:** I'd like to start with the process of looking at worms and tracing their lineages. When did this work begin?

**John Sulston:** It was mid-'70s, I think. I'm a bit vague because I did all sorts of different things after joining Sydney's [Brenner] group in 1969. One of those projects led me to being interested in the cell lineages, because I wanted to know where the dopamine-containing cells came from.

I was in the midst of a hobby project, a method for displaying catecholamines as bright fluorescent adducts. After some fiddling around, it worked and gave some beautiful patterns. It was clear that a very small subset of neurons contained dopamine and some contained serotonin. That was really no big deal.

However, what *was* potentially a big deal was that some of those cells appeared only after the embryo hatched. There had been a general view floating around, one of those urban myths, that there really wasn't any neural development after hatching. So I thought we should follow this up.

I started looking at these cells with Nomarski microscopes, of which there were several around the lab. I was lucky enough to stumble on a way to just look at them, without any fancy photography, and discover where the cells came from.

But then, we realized that other sections of the nervous system also developed post-hatching. The long and short of it was that I found myself able to watch dividing cells.

**JG:** At some point you moved from looking not just at neural cell divisions to looking at the whole thing.

**JS:** That's right. Just bit by bit. The very first lineaging was the ventral cord, a set of motor neurons that make the organism move in a sinusoidal wave, backwards and forwards.

Just after that, Bob Horvitz showed up. He was a hard-core molecular biologist. He thought all this zoology stuff was a bit of rubbish. He was fairly amazed that a guy was sitting there studying cell lineages.

It was quite an interesting rapprochement. Bob wrote it up as a nice thing for *Genetics* [1980] in which he described how this happened, how at first he was completely bemused, how if it didn't involve a scintillation counter it wasn't real science. He hadn't realized that this was really precise, really digital information coming out. Not sloppy stuff.

So *he* then became the driving force to push it to the next stage and do *all* of the post-embryonic lineaging. He did some, I did some.

**JG:** You talk of this period of a year and a half when you focused on the embryonic development, to complete the entire lineage.

**JS:** That was later. This is going back to the history and why people hadn't made progress with the fancy Nomarski microscopes and why I did. Because of my initial interests, I was looking at the larvae, which others had completely ignored.

**JG:** Because that stage was thought to be too late?

**JS:** Yes, because there was this view that the nervous system wasn't developing there anyway. Also, it was difficult because the worms are wriggling around. People were interested, but they killed them or anesthetized them, and of course, nothing happened because the worm is unhappy, or dead, or about to die. And the result was it was all fairly hopeless.

What I hit on was the idea of putting living worms down on an agar pad with some bacteria for them to eat, so they were happy! And although they were still moving, they weren't moving so fast that you couldn't follow the cells.

And this is what Bob Horvitz found me doing, just after I had completed the ventral cord work, and he joined in. And then we passed this technique—it seems a rather grand word for just looking at worms on an agar patch—on to people all over the world and it allowed them to start looking at post-embryonic development and make use of mutations and so on.

So there was great excitement about this. It was the little wonder of that particular year that suddenly we could see the cells. And it linked the work John White was doing on reconstructions of the neuroanatomy. He could, from the output of the lineage work, say which cell was which and correlate the position of the twig on the lineage tree and the type of motor neuron it was producing.

**JG:** And these reconstructions were by EM [electron microscopy]?

**JS:** Yes, and there was a guy named Nichol Thompson on whom everything hung because he was able to cut very long series of sections without losing *any*. It was a great deal of work, but it meant that people could go through those sections, make photographs, trace through the stack. Because there were no gaps, you could actually follow through the profiles of the axons.

So, then we wanted to know where these neuroblasts came from—to go back into the embryo. And this had been where people had thought to start because it was easy to have eggs survive in their hard egg case under the microscope. But the problem was that it was hard to follow the cell lineage in a ball of cells with really no phenotypic characters.

But there came pressure from the community to move forward on the embryo, because of a dispute. Someone had claimed to have the lineage of the intestine, and people were doubtful as to whether he got it right, so I was asked to arbitrate. So I ended up doing my first bit of embryonic lineaging using exactly the same method, just putting the egg under Nomarski optics and watching and drawing.

I can show you the patterns, if you want to see the way they're recorded.

**JG:** Yes, love to!

[John opens his small Sony PC to a record dated 5 June 1980. There are a series of colorful small drawings with circles and arrows, reminding me of an American football playbook. We delve into the drawing (Box 1).]

**Box 1: A Page from the Notebook of Sir John Sulston**

**JS:** Let me explain what you see on this page. This is a particular bunch of cells at a particular time. Here are the minutes during which I am watching it. Drawn every five minutes or so, over a span of about three hours. I broke it into stages—I followed a particular cell and its descendants.

And then I put these different bits together. It was based on the assumption that the lineage would be fixed. And, of course, as I did it, I checked to make sure that things really did always happen the same way, and indeed they did, apart from very rare cases where cells determine their fate by their interaction.

**JG:** So you've got red pens, green, blue, black pens, drawing quickly every five minutes. This is the kind of mundane thing I'm interested in.

**JS:** I thought it might amuse you. You've got red, green, black, blue, and purple on this one.

**JG:** This looks like Russian to me [as I pointed to a cell labeled “MStpaaapa”].

**JS:** These are the names of cells. MSt is one of the primary blast cells, and that means the posterior daughter, and the anterior daughter, and the anterior daughter and the anterior daughter and the posterior daughter and the anterior daughter. And there is a question mark! Wasn't sure if I got that right or not.

I should explain the colors. They are depth. Red is out the top, then down a bit is green, down a bit more black. This is the problem with movies, you get all these images at different depths and then you've got to try putting them together. Now, the drawing you're putting together over a limited area at the time. So I'm quite certain of what I'm looking at. However, I do have to follow about ten cells at a time.

**JG:** What are the hatch marks here?

**JS:** That is a cell death, one of the things that the worm has become rather well-known for. Programmed cell death was known about in mammals, but there were no handles on it. But because the worm has a fixed cell lineage and we knew which cells were going to die, we could look for mutants that change that pattern. The hatching means that the cell has gone very refractive—very bright—in Nomarski image.

**JG:** Are these the same color codes you used with the larvae?

**JS:** Yes.

**JG:** So you are actually thinking in color here.

**JS:** It's an approximately spectral range—red at one end, violet at another—so it is easier to remember. A cell higher in the focal plane is red.

These pictures have actually been quite interesting. In fact there is a whole bunch of these notebook pages in Kettle's Yard in Cambridge [“Lines of Enquiry: Thinking through drawing,” a fascinating exhibition by Barry Phipps http://www.kettlesyard.co.uk/exhibitions/archive/linesofenq.html].

**JG:** How did you settle on this kind of representation?

**JS:** Desperation.

The larva is more or less flat, and when a cell goes past another, you can keep on drawing. But what happens when a cell goes over another cell, oh dear, I get lost. So, I grabbed the nearest color pen and said, “Right, if it goes up it's red.” When I came to the embryo I was really equipped to code it. So this is a ball within a ball—ten cells sitting with several hundred cells at this stage.

**JG:** Love the squiggle—what is that all about?

**JS:** I made a mistake. And I started again.

**JG:** I see you are picking up from where you left off on the fourth of June. You had to find a new embryo and maybe wait around for it to be of the right age.

**JS:** I realized that I could come straight in if I picked the right stage embryo. And there is quite a business of doing this, sorting embryos and looking around for the one you want and finding the right orientation. You can pick up landmarks that are unique.

**JG:** So you started your day picking the one or two embryos that you were going to look at.

**JS:** That's right. Now on this one, I want to know what this particular cell is, and that is all I want to know. There is something called M1 coming out, sister to a cell death. There was something about that I didn't know, but here I can make a conclusion.

**Figure:**
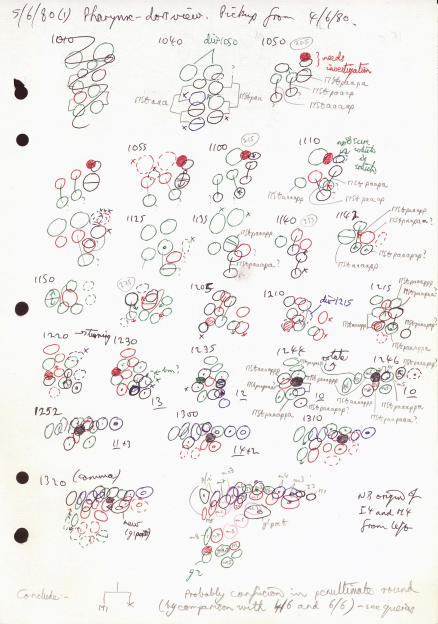

**JG:** Now would this page have been in a bound laboratory notebook?

**JS:** These are all loose leaf. If you had come to the house I could have pulled these things out. All my archives are there at the moment. They'll land up at the Wellcome Trust after a while. So if the house catches fire we'll lose them all. It's a bit doubtful how many of these you really want.

**JG:** Well, how many are there?

**JS:** Hundreds. This is really only of historical interest.

The technology for studying the embryo has improved. John White built a 4-D microscope, scanning up and down the whole embryo. And with nice software, you can ask for images in any series you want. And this really put embryonic lineage following on better footing. This came out about ten years after my work. Now there is yet another step, just happening in Bob Waterston's lab in Seattle. They are completely automating lineage mapping by green fluorescent protein-tagged histones.

**JG:** Did your mind ever wander while you were doing this?

**JS:** You have to be quite focused. I did really button it down. You don't need to draw many pictures when the nucleus is just changing its morphology, but you do have to be really on the ball when the cell goes through its anaphase and telophase because that is what you must follow in order not to be confused.

**JG:** Were you in a room by yourself?

**JS:** Oh, yeah.

**JG:** For all these kinds of experiments?

**JS:** I always was alone. I find it impossible if there is distraction.

I remember there was a little bit of conflict about this. Some of the others rather wanted a microscope room, and that is good because it's air-conditioned. In order to keep watching these you must keep the temperature down to about 20 °C. It's very easy to overheat [the worms] when you are shining bright light down and the slides get hot. Above 25 °C they start to get very unhappy.

And in a lot of labs like Bob Horvitz's lab at MIT, there is a whole row of microscopes, and that is true of anyone now who does this kind of thing. But I noticed, when I visited there, that they really don't talk.

But I remember at LMB [Laboratory of Molecular Biology, Cambridge], people would come in and say, “Oh, look what's happening,” and they really wanted to talk about it, and I'd completely lose it! So I said, “Look, I have to have my own room for this.” People were feeling, “Oh, the poor guy is a bit fussy,” but I got my way, and otherwise I wouldn't have been able to do it.

**JG:** When was it that you got your own room?

**JS:** When Bob and I seriously started to look at post-embryonic lineage. I think I just made myself sufficiently obnoxious.

**JG:** So you didn't listen to music or anything when you did this?

**JS:** One thing I did do with the embryonic lineage, I solved the Rubik's cube from scratch. I also made some Archimedean solids, the regular polyhedra. I made them out of thin card and glued them together. So I did find myself little distractions, but obviously things that didn't engage me.

**JG:** Where was Bob?

**JS:** He was in the room next door. He was doing various other things as well, genetics, while I was doing just solid lineaging. We worked on the first mutants that affected the cell lineage. It was the beginning, I think quite a nice catalog.

Bob is a very thorough man. I remember he made an enormous list of about 150 psychoactive drugs to try on the worm. And any time something happened we would work on it together. I did more of the following up, so in addition to the wild-type notebooks, there are notebooks full of the mutant images.

**JG:** What do you think it is about *you* that made you particularly good at this lineage work? Not everyone would have the patience and the focus to go through this.

**JS:** Well, I don't think I had anything else much to do at the time.

All of my scientific projects have started as hobbies, something to do in the evenings maybe, and then at some point you find you are taking it seriously and it becomes your day job. It was this business of constantly trying things out and seeing what you *can* do.

Why me? I don't know. I enjoyed looking at the Nomarski objects. Actually, I think they are awfully beautiful. I still find it amazing that you have this optical trick that allows you to visualize what is only a transparent image, after all. Because of the cross-polarized beams, you image the refractive index. The detail you see! I could watch it forever.

And then there was the group thing. I emphasize Bob, but the whole group was involved. And there was this feeling that the lineage was something that people really wanted.

That's the other thing—I actually quite like to please.

**JG:** You never entered the professorial track, but remained a staff scientist at the LMB. Did that suit your temperament?

**JS:** Absolutely. It was a very good fit for me. If there was something that I thought was important and worth doing, I could just focus on that, and the only pressure was from family life. It meant you had time to sort all this out and not feel the pressure that you had to cut corners or guess.

The negative is that we don't do much teaching. I feel in a way we're not such good citizens because teaching is important. Also, we didn't have independence. As long as I was at the LMB working on worms, Sydney was my boss.

**JG:** Sitting there in a chair, focusing on this process—it has the feeling to me of a meditative practice. Did you have a kind of spiritual experience?

**JS:** I would only use the word spiritual in the loosest sense. I use the word beauty straightaway, and I think that touches on it. Enjoying this sort of thing is certainly part of humanity. And I'm the secular humanist kind of person, who thinks that we have a love of beauty built into us.

I feel the same way, hiking in Scotland in Ben More. [He points out his laptop screensaver with his photo of the vista, stippled with icons].

**JG:** And how marvelous to watch something that nobody has ever seen before.

**JS:** Oh yes! The most exciting thing to do is to go somewhere where nobody has been before. It was *hugely* exciting, looking at those cells dividing for the first time and knowing that I could see, I could find out. It's true all the way through science.

Actually you hold it to yourself [speaking in a hushed voice, as he folds his arms across his chest] when you've got something nice, but then within a few minutes you've got to rush out.

**JG:** What were the times you rushed out?

**JS:** Seeing those first cells divide! Because the previous work looking at fixed cells just hadn't gone anywhere. No one had managed to see anything. But I was just sitting there and suddenly I knew it was all open. Because I could see that first cell divide and I knew I could see its daughter divide.

**JG:** Did you wait for its daughter to divide before rushing out of the room?

**JS:** Of course! The very first viewing I carried on right to the end. I was entranced. And those divisions are quite quick. Within an hour, it's already beginning to swell up to the next stage.

**JG:** But another moment might have been when you saw the first cells dying off.

**JS:** That was a rather slower dawning of what was going on. At first, I didn't know what to make of it at all. There must have been some discussion with others about what on earth was happening. I don't know if I or someone else first realized that this might have been a cell death. I just knew that something strange was going on, and this thing had disappeared, so obviously it was a cell death. But I probably wasn't sufficiently well-read to know the background to this and to know about cell death being a programmatic feature in development.

**JG:** Let's tear ourselves away and move forward to the nematode genome work.

**JS:** At the end of the embryonic lineage work, I didn't know what to do. I wrote that up and it was done. And everybody said, “Well, now of course you do mutants.”

But it was a bit competitive by then. There were all sorts of people out there doing it, including my ex-student. I wasn't sure I really wanted to go and compete. It's a bit of a mingy thing to say, but there you go.

I like to do things on my own, you see. I don't like being in the middle of buffaloes on the plain kicking up the dust. So I was looking around for something else to do more on my own.

A rather dramatic flash came when I heard a seminar by Matt Scott describing the *Antennapedia* walk, working out the map of the region of *Drosophila,* and I said, “We've got to do this [in worms], but not just one bit—the whole thing!” Because at that point there was this absolute bottleneck with the worm people.

I remember discussing this with Bob Horvitz and Jonathan Hodgkin. And I remember being quite worried. It wasn't at all clear to me that the worm was going to make it! We lacked some of the perquisites of the fly, the polytene chromosomes in particular. The fly came with this built-in physical map, which the worm didn't. And one of the consequences was that our students were spending years, their whole Ph.D. theses, isolating a gene. It was awful. John White complained about this. He said the seminars were deadly, mind-numbing to sit and listen to each student explain how they had failed to clone their gene.

So after hearing Matt speak, I came back and talked to people about how we're going to do this. And I said “Look, we're going to start.” So I was given some space in the new bit at the LMB to do this.

**JG:** Did you ever imagine that it would turn into what it eventually became?

**JS:** No, absolutely not. I just saw it as a specific problem and it was very much oriented on the map.

**JG:** And the recovery of clones.

**JS:** Yes. Just sort the thing out and have the clones lined up in the freezer, so people could take them and have a reasonable correlation between the physical and genetic map. That was it—that was to be the project for the '80s.

That really was a big change in my life, and that led directly beyond, not only to the worm sequence, but then to the human. As night follows day, to this place being built. And everything that happened in the '90s around here.

**JG:** Was Alan Coulson just leaving Sanger's lab at that time?

**JS:** That was the other very good thing that happened to me, and probably without that, it would not have worked. Alan chose to join me when Fred Sanger retired. I had already been working, rather ineffectively, on it. Alan is superb at making things work and parallelizing. So we had a hell of a fine collaboration and things started to move immediately when he came.

**JG:** And it sounds as though you were engaged with this new project as much as you were with the old one.

**JS:** Exactly. It was an important problem. And we needed it [the map].

What I did for much of the '80s was make the libraries, for some reason, because Alan was getting on with the biochemistry. But the thing I mainly did was to learn computer programming. I took that up and wrote all the software. I had no intention of doing that, but there was no one else to do it, and we needed it urgently. So one weekend, I said, “I'm just going to start writing Fortran.” Roger Staden started me off and it snowballed into this huge program, very unprofessionally written, but it worked.

When we had done two or three years of this and more or less exhausted the approach we had, which was looking at cosmids, we became aware there were gaps. Bob Waterston came and we all worked on this. From the mid-'80s this was a formal and equal collaboration. And it was resolved by Bob's going back and using the YAC [yeast artificial chromosome] technology that had just been developed in Maynard's [Olson] lab. And suddenly we had the whole map.

**JG:** OK, let's fastforward to more recent history at the Sanger Institute. Why did you decide to step down as the director in 2000?

**JS:** Well, the decision was made in 1998, and the people I'd worked with weren't terribly happy about it—a bit rocking the boat.

We were just a bunch of amateurs, the seven of us on the board of management, and the staff did get very unhappy after about three years when the numbers went up to about 100, and the Institute wasn't being running along stable, robust, and predictable lines. So we had some management training that was quite interesting and extremely effective.

We all underwent a bit of psychoanalysis of our various characteristics. When it came to the results from me, the facilitator said “Well, you're the sort of person who gets there in the end by a very muddled route and you end up emerging backwards through the hedge covered in bits of glass.” I was rather flattered; I thought he got that about right!

And that's back to your question about doing things differently. If you worry too much about how you're going to do something, you don't do anything.

**JG:** So, back to leaving the directorship . . .

**JS:** It was like leaving the lineage work. I felt I had done what I could, and I felt quite strongly that it was going to plateau, and I wasn't a proper director, in the sense of really *enjoying* running things. I didn't have yet another big project that would move things on. It would become more of an administrative thing. What Allan [Bradley, current WTSI director] has done is to hire a lot of new faculty doing a lot of different things and the place has become much more like a university department with a lot of the high-throughput stuff still here.

I really can't run a department in this way. It was a single project thing for me.

**JG:** Ah, you are focused.

**JS:** But I had enough of a sense of preservation of the Institute that I was always enthusiastic about having multiple things going on.

**JG:** When you stepped down, did you want to relinquish the administrative responsibility but continue with the genome project?

**JS:** I didn't have a particular thing in mind. But at that point, there was still too much to do. In 1998 we had published the worm in incomplete form and still had a lot to do on closure. The human genome was just reaching its crescendo. And I told people I was not stepping down from that. I worked as hard as ever on the worm and on the human right through 2001, and then things began to fade a bit. And by that time I'd got the notion of writing a book with Georgina [Ferry].

So somehow, I never really considered what I would do next because the time was full.

**JG:** I've also heard that when you win a Nobel Prize your time is no longer your own.

**JS:** It's very demanding. You have to sort it out. It's impossible.

**JG:** Do you still have a lab here?

**JS:** No. A couple of years ago I became Emeritus with the Wellcome Trust. I pop in at least once a week, often on Sunday morning when it's quiet. Sometimes with a specific intent or a meeting, or I'll meet people in passing. I use the library quite often. I still like using books.

**JG:** Looking back, was there one period that sticks out as being the most joyful or the most productive or the most intellectually exciting?

**JS:** I wouldn't discriminate. Something I want to emphasize is how distinct they are, really. My postdoctoral work on prebiotic chemistry with Leslie Orgel was hugely exciting, and before that I enjoyed being a student with Colin Reese just making some of the early oligonucleotides. But there is this sense that I, probably more than most, have jumped from one thing to another.

**JG:** Yes, but with a rather slow periodicity. You couldn't be faulted for leaving a project before it was completed!

**JS:** Yes, a classic seven years. Well, I suppose the genome is rolled itself together into two lots of seven years.

**JG:** When I read *The Common Thread,* I was struck by the image of you and the public human genome project as a ship under a state of attack. That you were thrust into a position of having to suddenly defend yourself and your approach against the assault of entrepreneurial javelins, and to work even harder. What a huge waste of time and resources the battle caused!

**JS:** It was hugely complex. And that was exactly why things changed again. And any immediate thought of restarting a group went out of the window! It was a waterfall of things happening! And then, all I wanted to do was to write an account of it afterwards and talk about it because I thought it was important.

But a lot of people say, “Well, this is the way the world is. You should know that!” And that I find very depressing. There is a sense in which we are struggling to come to terms with a very rapacious free market, in all sorts of areas. And this little particular area is what I was trying to express in the book.

There is a lot of rewriting of history now, but it was just like that—the assault with javelins.

What I've never been willing to agree to is that somehow or other it helped! The standard story, because everybody wants peace, is to say, “Well, yes this competition did accelerate things.”

I think that is absolutely wrong. All it did was to speed up getting this *fake* release of the draft sequence, which was 90% complete. It was a political deal. It was an election year [2000]. The White House had really become unhappy about what was going on. It was a silly deal, but it meant peace.

**JG:** Have you met Craig Venter?

**JS:** The last time I saw him was at the Gairdner. One of the things we've had to do since this is to all go and get awards together. What else can you do? You can't be in a state of perpetual warfare. And what you said about it being a huge waste of time is absolutely right.

**JG:** Do you think Americans are most aggressive about privatization? Do you think such a thing could have happened in England?

**JS:** Well, we have rapacious entrepreneurs here, too, but America is bigger and has the world's richest corporations.

Of course, there was a battle within the US, which was almost divided down the middle—the neoconservative and Democrat wings. Yes, because the resources are there, I think these battles are more likely to be fought out in America.

But of course, increasingly so, they are also fought on the global campus, and that's the other reason why I think this is so important. We are wasting time, I think, to get globalization right, and people ought to be paying more attention to stories like this because it shows ways that things can be run stably in the future and ways in which they can't.

If you have unbridled competition as the basis for international relations, then we are going to die. Because we don't have the basis for sorting out the environment or people's lives or anything in a more ethically advantageous way. If everything is done through the World Trade Organization, and everything is done for who can get the most revenue, we're sunk.

**JG:** Were you thinking, when you made plans to retire, that you were just going to end it [your career]?

**JS:** No, I knew I wasn't just going to end it, because I had too much to do. What I hadn't planned on was what it might lead to. I've dealt with how things have evolved and haven't started new ones. That was not a plan.

**JG:** Can you articulate your role now?

**JS:** I am occupied with various small attempts to lubricate the interface between the science of genetics, human genetics, and the public—all of us.

I'm the vice-chairman of the Human Genetics Commission, a part of the Department of Health in the UK. The aim is to have a body of experts, about twenty of us in various areas—industry, science, nursing, consumer affairs—all people with a stake in genetics in general. The thing I'm most concerned with is genetic equity. Simply nondiscrimination, because it's a real issue.

Another thing I'm doing informally is speaking in support of various NGO activities in Geneva. I'm interested in fair trade. Medecins sans Frontieres and Oxfam both run campaigns on this which I've supported. There is the Drugs for Neglected Diseases Initiative, which I support.

There is also an active campaign going on in WIPO [World Intellectual Property Organization] to bring a development agenda. The background to all this is immensely complex. In the WTO [World Trade Organization], the TRIPS agreement [Trade Related Aspects of Intellectual Property Rights] is coming to a head and is forcing all but the very poorest countries to sign up for a high level of enforcement of law as practiced by the G8 countries. What this means is that India will no longer be able to produce generic drugs freely. It will greatly increase the powers of the major corporations.

One must not be cavalier and say, “This is all bad and we must sweep it away,” but unquestionably the current system we have for funding medical R and D leads to production of pharmaceuticals only for the rich markets.

**JG:** What about the Gates Foundation?

**JS:** The Gates Foundation is the *only* major source of revenue for these initiatives right now. And all the public/private partnerships are dependent on Gates money at the moment.

It's still not enough and it's not sustainable enough, even though Warren Buffett has just contributed his money as well. So perhaps the world will be run by rich men's charities. I find that rather uncomfortable. Of course, the reason we are here in this building and sequencing the human genome is because of pharmaceutical stock from the Burroughs Wellcome Foundation.

So things are a mess. What we're up against is whether we can have real intergovernmental support for drug development that will not depend on the marketplace. *PLoS Biology* [www.plosbiology.org] published a new concept for an international treaty on biomedical research. Jamie Love, who runs his own NGO called CPTech in Washington, and Tim Hubbard, who is here, have put together a scheme for an international accord by which countries contribute to a global healthcare R and D fund. This can be done through the free market if people wish, but equally could be done in an open access sort of way, through institutions. It would be much more transparent. Above all it would allow us to devote funding not just to the revenue-earning things in the rich markets. But we could start to address the neglected diseases.

It would generate an enormous worldwide fund, far bigger than the Gates Foundation. This is the kind of proposal that really raises the ire of the pharmaceutical representatives.

It would be a hugely cohesive thing in the world if we were able to move towards a global health system. But I fear that the force is going the other way. The things I've heard the representatives of the pharmaceutical manufacturers say in Geneva—the blatant, vicious manipulation of figures to prove their case! And it sounds silly—but just the rudeness!

**JG:** The arrogance?

**JS:** Yes, exactly. They say: “You're wasting your time. Nothing will ever change.” How appalling!

**JG:** Maybe these kinds of problems make the human genome project seem not so complex!

**JS:** When I give speeches to people, I do tend to end up as a prophet of doom, saying we really maybe won't live another century. And one of the ways we may *not* do it is to continue along this path.

**JG:** On a much lighter note, I have a final question. I will never meet a knight again, so . . .

**JS:** There are quite a lot of them about, actually.

**JG:** There are?

**JS:** This is quite interesting. For a long time the guys in LMB didn't accept knighthoods. Max Perutz, for example. And not long before he died, I asked him about this. He said he didn't because it would set him apart from the staff.

Now, it's important to look at background on this. Those guys, when they set up the LMB in their new building, set up a single canteen. More or less contemporaneously, I was in research in the Chemistry Lab, and there were three canteens—one for the faculty, one for the students, and one for the technicians. Absolute class distinction, and that is the way it was always done.

So, in that context, Max didn't want anybody to be put in a position of uncertainty about how to address him. He was just “Max.”

Max was not a sort of hale fellow. He was very quiet and quite formal, so people might have been inclined to give him his title. He was a very properly spoken kind of guy. And it was wonderful to learn that underneath was this well of egalitarianism.

**JG:** Fascinating.

**JS:** It is a real problem with knighthoods.

**JG:** Sorry, I must laugh, sounds like a problem with putting on your armor or something!

**JS:** The reason I said that there are quite a lot of them is because there are! It is not the top honor. You also have MBE, OBE, CBE, then knight, then various higher ranks.

**JG:** You just jumped in at knighthood?

**JS:**? Yes. People put you up for these things. You just get a letter one day saying you are being offered a knighthood, are you going to accept it? There is just this curious convention that with the knighthood you can put “Sir” in front of your first name, not your second name.

The whole thing is absolutely ridiculous, really. But I was persuaded that it was quite a good thing for science to be recognized in this way in this country. Aaron Klug was the first one to accept it at the LMB. And he began to talk to us about how accepting knighthood was good for science. Otherwise science was the poor relation and business and arts were recognized. Of course, some people ask how could I accept this—republicans and the [Manchester] Guardian—and I say this has nothing to do with the Queen. Although you do meet the Queen.

**JG:** You do?

**JS:** Yes, the sword on the shoulder. It's all pretty odd. So we either cold-shoulder it, or we say scientists are just as good as anybody else.

**Figure:**
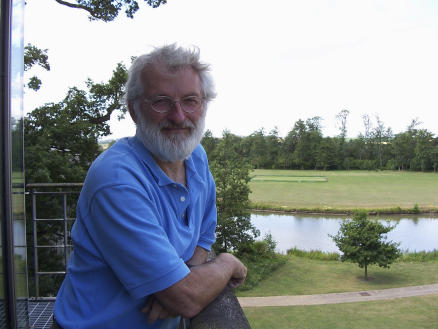
Sir John Sulston

